# Risk of cardiovascular toxicity with combination of immune-checkpoint inhibitors and angiogenesis inhibitors: a meta-analysis

**DOI:** 10.3389/fcvm.2024.1309100

**Published:** 2024-02-02

**Authors:** Alessandro Inno, Antonello Veccia, Giorgio Madonia, Alvise Berti, Roberto Bortolotti, Lorena Incorvaia, Antonio Russo, Orazio Caffo, Stefania Gori

**Affiliations:** ^1^ Medical Oncology, IRCCS Ospedale Sacro Cuore Don Calabria, Negrar di Valpolicella, Italy; ^2^ Medical Oncology, Santa Chiara Hospital, Trento, Italy; ^3^ Deparment of Surgical, Oncological and Oral Sciences, Section of Medical Oncology, University of Palermo, Palermo, Italy; ^4^ Center for Medical Sciences (CISMed), Department of Cellular, Computational and Integrative Biology (CIBIO), University of Trento, Trento, Italy; ^5^ Rheumatology Unit, Santa Chiara Hospital, APSS, Trento, Italy

**Keywords:** immune checkpoint inhibitors, angiogenesis inhibitors, multikinase inhibitors, cardiovascular toxicity, hypertension, stroke, myocardial infarction, pulmonary embolism

## Abstract

**Introduction:**

Combinations of immune checkpoint inhibitors (ICIs) and angiogenesis inhibitors (AIs) have been investigated for the treatment of several tumor types. Both ICIs and AIs may lead to cardiovascular adverse events, and their combination may potentially increase the risk for cardiovascular toxicity. In the present meta-analysis, we aim to assess the cardiovascular toxicity of ICIs plus AIs vs. AIs alone. Secondary objectives are non-cardiovascular adverse events and efficacy.

**Methods:**

Systematic review was performed according to PRISMA statement. Phase II and III randomized clinical trials were identified by searching the MEDLINE/PubMed, Cochrane Library and ASCO Meeting abstracts, from inception to June 2022. The pooled risks for overall response rate (ORR), 1-year progression-free survival (PFS), adverse events (AEs), immune-related AEs, (irAEs), hypertension, and vascular events defined as stroke, myocardial infarction and pulmonary embolisms, were calculated.

**Results:**

In terms of cardiovascular toxicity, we found higher risk for severe hypertension among patients treated with ICIs plus AIs as compared with those receiving AIs (OR 1.24, 95% CI: 1.01–1.53), but no significant difference was found for any-grade hypertension, and for vascular events. There was also no difference in terms of overall AEs, whereas the incidence of irAEs was increased in the ICIs plus AIs arm, as expected. In terms of efficacy, ICIs plus AIs achieved better ORR (OR 2.25, 95% CI: 1.70–2.97) and PFS (HR 0.49, 95% CI: 0.39–0.63) as compared to AIs alone.

**Conclusion:**

The addition of ICIs to AIs significantly increased the risk of high-grade hypertension, but not that of acute vascular events.

## 1 Introduction

Immune checkpoint inhibitors (ICIs) have deeply changed the landscape of cancer treatment (1). By unleashing the brakes on the immune system, ICIs improve the effectiveness of immune response against cancer cells. However, they can lead to the development of immune related adverse events (irAEs), that may potentially affect any organ or system, including cardiovascular system (2, 3). Particularly, ICIs are associated with myocarditis, non-inflammatory heart failure, arrythmias and conduction disease, pericardial disease, vasculitis, myocardial infarction, and atherosclerosis progression (3–5).

There is a strong rationale for combining ICIs with angiogenesis inhibitors (AIs). In fact, the aberrant tumor vasculature represents a challenging barrier for T-cells to overcome in order to infiltrate cancer deposits and elicit anti-cancer cytotoxic activity; furthermore, vascular endothelial growth factor (VEGF), secreted by cancer cells to increase angiogenesis within a hypoxic tumor tissue, has inhibitory effects on cytotoxic T-cells and contribute to maintain an immune suppressive tumor microenvironment (6, 7). By inhibiting angiogenesis, AIs normalize tumor vessels and modulate the tumor immune microenvironment, thus potentially enhancing the action of ICIs. However, AIs also have cardiovascular toxicity in terms of hypertension, cardiac ischemia, cardiac dysfunction, and arterial thromboembolism (8). Retrospective data suggest an increased risk for cardiovascular events with the combination of ICIs plus AIs over single agents (9).

The main objective of this systematic review and meta-analysis is to assess the cardiovascular toxicity of ICIs plus AIs compared to AIs alone. Secondary objectives include non-cardiovascular toxicity and efficacy in terms of response rate and survival.

## 2 Methods

### 2.1 Systematic review

Systematic review was conducted according to the PRISMA statement (10). A comprehensive search of MEDLINE/PubMed, Cochrane Library and ASCO Meeting abstracts, from inception to June 2022 was performed using the terms “ipilimumab”, or “tremelimumab”, or “nivolumab”, or “pembrolizumab”, or “atezolizumab”, or “durvalumab”, or “cemiplimab”, or “avelumab”, and “bevacizumab”, or “axitinib”, or “lenvatinib”, or “sorafenib”, or “regorafenib”, or “sunitinib”, or “aflibercept”, or “pazopanib”, or “nintedanib”, or “cabozantinib”. Eligible studies were phase II or III randomized clinical trial comparing a combination of ICIs and AIs in the experimental arm and AIs in the control arm. Trials including chemotherapy regimens (in the control and/or experimental arm) were eligible. Trials were considered eligible independently of type of solid tumor, setting and line of therapy. Only articles published in English language were included. In case of duplicates or updates of already included trials, only the earliest publication for each trial was considered, since it usually was the one with the most complete safety data reporting. For trials including more than one experimental arm, only those including ICIs plus AIs were considered, whereas other experimental combinations were excluded from the analysis.

Two authors (AI and GM) collected data independently and any discrepancies were resolved by consensus with a third author (AV). Data on trial characteristics, study population, main outcomes and adverse events were collected from each study.

### 2.2 Definition of outcomes

Data on adverse events (AEs), irAEs, hypertension, acute vascular events, overall response rate (ORR), progression-free survival (PFS), and overall survival (OS) were collected. For the purposes of the present meta-analysis, acute vascular events were defined as stroke, myocardial infarction, and pulmonary embolism (PE), which was declared in all the studies. Severe AEs, irAEs, hypertension and acute vascular events were defined as grade ≥3 according to the National Cancer Institute Common Terminology for Adverse Events (CTCAE) version 4.0 or 4.03, as reported in each trial.

### 2.3 Statistical plan

The pooled risks for ORR, 1-year PFS, AEs, irAEs, hypertension, and acute vascular events were expressed as the total number of cases for each of these outcomes divided by the total number of subjects treated with the same type of treatment from different trials. The experimental arm and the control arm of each trial were compared to estimate the relative risks computed as the odds ratio (OR) for AEs, irAEs, hypertension, acute vascular events and ORR, whereas hazard ratio (HR) was used for PFS. Sensitivity analyses were performed.

The Mantel–Haenszel random effect method was used to obtain the pooled OR and the corresponding 95% confidence interval (CI). We used the X2 Cochran Q test to detect heterogeneity across the different trials. All the analyses were performed with Review Manager version 5.3.

## 3 Results

### 3.1 Description of included studies

**Figure 1:**
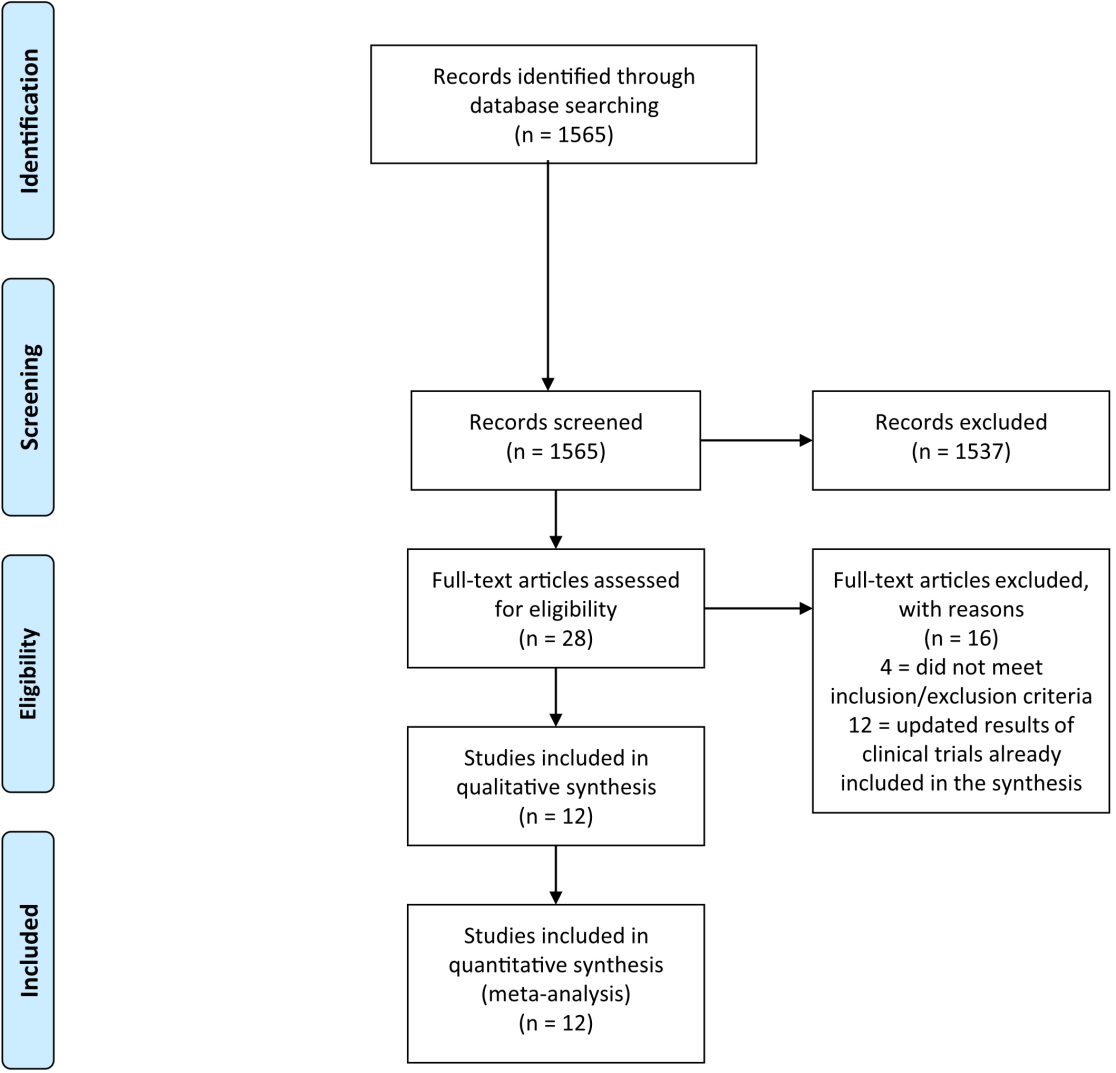
PRISMA flow diagram of included studies.

The systematic literature search returned 1,565 records. After the exclusion of 1,537 non-relevant records, 28 potentially eligible studies were considered (Figure 1). Among them, 4 were excluded because the control arm did not include AIs, and 12 because they were duplicates/updates of previously published trial results. At the conclusion of the selection process, 12 trials were included in the meta-analysis, with a total of 8,124 patients (4,159 in the experimental arms and 3,965 in the control arms) (11–22).

The main characteristics of the studies are reported in Table 1. Ten studies were phase III randomized trials, whereas the remaining 2 studies were phase II randomized trials. Eight trials were open-label, 4 trials were placebo-controlled. Trials enrolled patients with renal cell carcinoma (RCC, *n* = 6 trials), non-small cell lung cancer (NSCLC, *n* = 2 trials), hepatocellular carcinoma (HCC *n* = 1 trial), colorectal cancer (CRC, *n* = 1 trial) ovarian cancer (*n* = 1 trial), cervical cancer (*n* = 1 trial), at advanced/metastatic stage, mostly in the first-line setting. In the control arms, AIs were tyrosine kinase inhibitors (TKIs) in 7 trials (sunitinib, *n* = 6 trials; sorafenib, *n* = 1 trial), and bevacizumab in 5 trials. In the experimental arms, ICIs were atezolizumab (*n* = 6 trials), pembrolizumab (*n* = 3 trials), nivolumab (*n* = 2 trials) and avelumab (*n* = 1 trial), whereas AIs were TKIs in 4 trials (axitinib, *n* = 2 trials; lenvatinib, *n* = 1 trial; cabozantinib, *n* = 1 trial), and bevacizumab in 8 trials. Chemotherapy was included in both experimental and control arms of 5 trials, whereas in 7 trials both arms were chemotherapy-free.

**Table 1:** Studies included in the systematic review and meta-analisis.

Author, year	Trial	Phase	Cancer type	Stage	Setting	Experimental arm (*n*)	Control arm (*n*)	Median FU (months)	ORR (%)	PFS (months, HR)
Choueiri et al., 2021 (11)	CheckMate 9ER	3	RCC	Advanced	1st line	nivo + cabo (323)	sunitinib (328)	18.1	55.7 vs. 27.1	16.6 vs. 8.3, 0.51
Colombo et al., 2021 (12)	KEYNOTE-826	3	Cervical	Advanced/ recurrent	1st line	pembro + bev + pbCTx (308)	placebo + bev + pbCTx (309)	22	65.9 vs. 50.8	10.4 vs. 8.2, 0.65
Finn et al., 2020 (13)	IMBrave150	3	HCC	Advanced	1st line	atezo + bev (336)	sorafenib (165)	8.6	27.3 vs. 11.9	6.8 vs. 4.5, 0.59
McDermott et al., 2018 (14)	Immotion 150	2	RCC	Advanced	1st line	atezo + bev (101)	sunitinib (101)	20.7	32 vs. 29	11.7 vs. 8.4, 1.0
Mettu et al., 2022 (15)	BACCI	2	CRC	IV	Pretreated	atezo + bev + cape (82)	placebo + bev + cape (46)	20.9	8.5 vs. 4.4	4.4 vs. 3.6, 0.75
Moore et al. 2021 (16)	IMagyn050	3	Ovarian, fallopian tube, peritoneal	III/IV	Neoadjuvant/1st line	atezo + bev + cbdca + pac (651)c	placebo + bev + cbdca + pac (650)	19.9 / 19.8	93 vs. 89	19.5 vs. 18.4, 0.92
Motzer et al., 2020 (17)	JAVELIN Renal 101	3	RCC	Advanced	1st line	pembro + lenvatinib (355)	sunitinib (357)	26.6	71 vs. 36.1	23.9 vs. 9.2, 0.39
Motzer et al., 2021 (18)	CLEAR	3	RCC	Advanced	1st line	ave + axitinib (442)	sunitinib (444)	12 / 11.5	51.4 vs. 25.7	13.8 vs. 8.4, 0.69
Rini et al., 2019, (19)	KEYNOTE-426	3	RCC	Advanced	1st line	atezo + beva (454)	sunitinib (461	24	37 vs. 33	11.2 vs. 8.4, 0.83
Rini et al. 2019, (20)	IMmotion151	3	RCC	IV	1st line	pembro + axitinib (432)	sunitinib (429)	12.8	59.3 vs. 35.7	15.1 vs. 11.1, 0.69
Socinski et al., 2018 (21)	IMpower 150	3	Non-squamous NSCLC	IV	1st line	atezo + bev + cbdca + pac (400)	bev + cbdca + pac (400)	20	63.5 s 48	8.3 vs. 6.8, 0.61
Sugawara et al., 2021 (22)	ONO-4538-52/TASUKI-52	3	Non-squamous NSCLC	IIIB-IV	1st line	nivo + bev + cbdca + pac (275)	placebo + bev + cbdca + pac (275)	13.7	61.5 vs. 50.5	12.1 vs. 8.1, 0.56

Atezo, atezolizumab; bev, bevacizumab; cape, capecitabine; cbdca, carboplatin; HCC, hepatocellular carcinoma; HR, hazard ratio; nivo, nivolumab; NSCLC, non-small cell lung cancer; ORR, overall response rate; pbCTx: pac, paclitaxel platinum-based chemotherapy; pembro, pembrolizumab; PFS, progression-free survival; RCC, renal cell carcinoma.

Three trials included more than one experimental arm: the phase II IMmotion150 trial compared atezolizumab alone, atezolizumab plus bevacizumab, and sunitinib in patients with advanced RCC (14); the phase III CLEAR trial compared lenvatinib plus pembrolizumab, lenvatinib plus everolimus, and sunitinib in patients with advanced RCC (8); the phase III IMpower150 compared atezolizumab plus carboplatin plus paclitaxel, bevacizumab plus carboplatin plus paclitaxel, or atezolizumab plus bevacizumab plus carboplatin plus paclitaxel in patients with advanced non-squamous NSCLC (21). For the purposes of the present meta-analysis, only experimental arms based on combinations of ICIs plus AIs and control arms based on AIs were considered (i.e., atezolizumab plus bevacizumab vs. sunitinib in the IMmotion150 study; lenvatinib plus pembrolizumab vs. sunitinib in the CLEAR study; atezolizumab plus bevacizumab plus carboplatin/paclitaxel vs. bevacizumab plus carboplatin/paclitaxel in the IMpower150 study).

### 3.2 Cardiovascular toxicity

**Figure 2:**
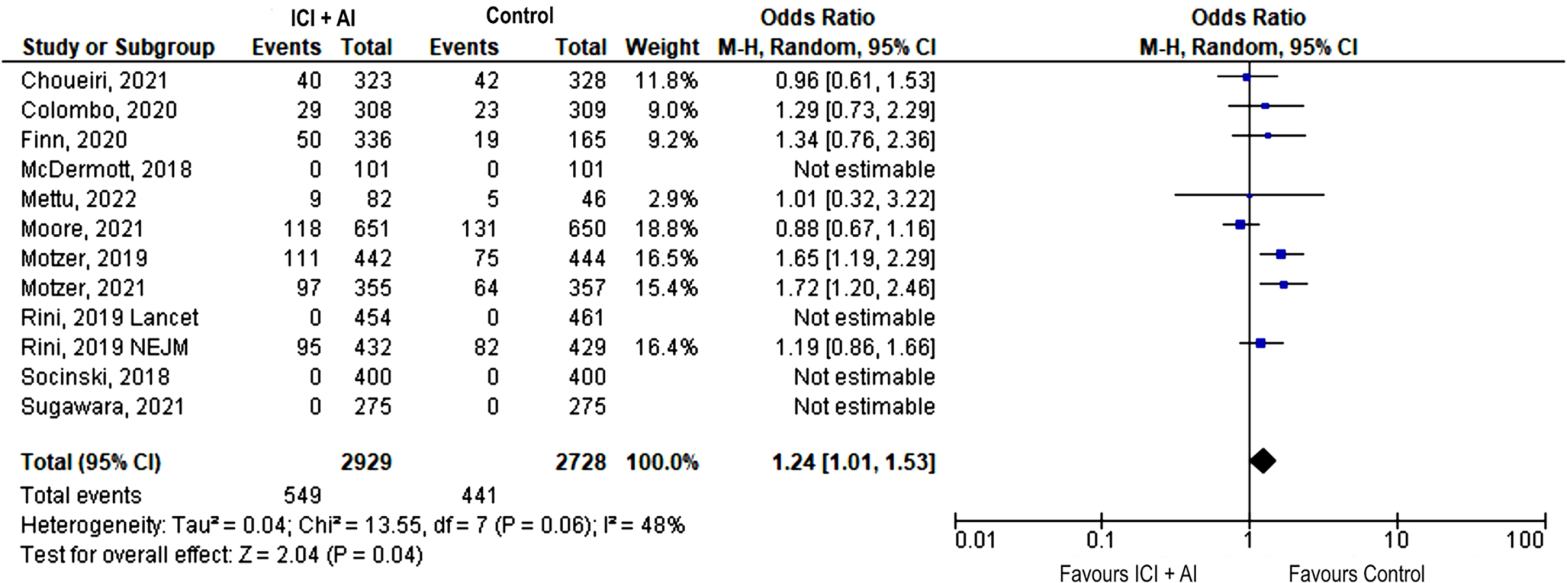
Forest plot showing odds ratio for severe (grade ≥ 3) hypertension adverse events for the 12 studies that explicitly reported the number of severe hypertension adverse events, respectively, by ICIs + AIs vs. control. The risk ratio for each adverse event is represented by a square, and the horizontal lines crossing the squares represent the 95% confidence interval (CI).

The most frequently reported event across trials was hypertension. Although there was no significant difference in terms of any grade hypertension (38.9% vs. 36.6%, OR 1.17, 95% CI: 0.91–1.50, *p* = 0.23), the incidence of severe hypertension was significantly increased among patients receiving ICIs plus AIs (18.7% vs. 16.2%, OR 1.24, 95% CI: 1.01–1.53, *p* = 0.04) (Figure 2).

**Figure 3:**
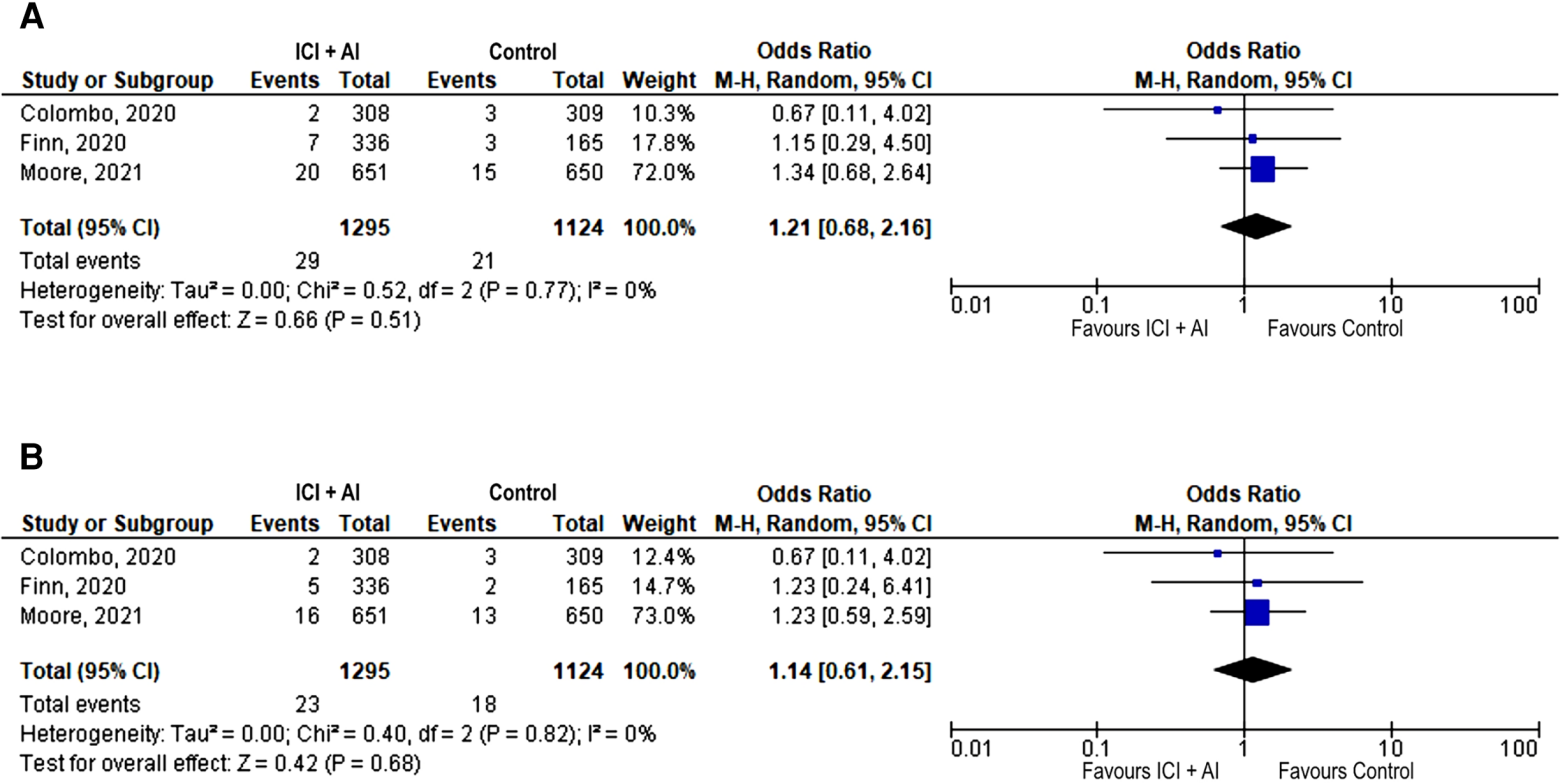
Forest plot showing odds ratio for all reported (**A**) and severe (grade ≥ 3) (**B**) acute vascular events in the 3 studies that explicitly reported the number of total and severe acute vascular events, respectively, by ICIs + AIs vs. control. The risk ratio for each adverse event is represented by a square, and the horizontal lines crossing the squares represent the 95% confidence interval (CI).

Only 3 trials reported data on acute vascular events, with a low number of events (*n* = 50), that were severe in most cases (*n* = 41, 82%) (12, 13, 16). There was no significant difference between the ICIs plus AIs and AIs arm in terms of incidence of acute vascular events, neither any grade (2.2% vs. 1.9%; OR 1.21, 95% CI: 0.68–2.16, *p* = 0.52) nor severe (1.8% vs. 1.6%; OR 1.14, 95% CI: 0.61–2.15, *p* = 0.68; Figures 3A, B).

PE accounted for most of the reported acute vascular events, with no significantly increased odds observed among patients receiving ICIs plus AIs compared with those receiving AIs alone, neither for any grade (OR 1.27, 95% CI 0.65–2.46, *p* = 0.48) nor for severe grade (1.8% vs. 1.5%, OR 1.34, 95% CI: 0.64–2.79; *p* = 0.44). Other acute vascular events occurred less frequently: the incidence of stroke was 0.4% in both arms (ICIs plus AIs and AIs), as reported in 3 trials (12, 13, 16), and that of myocardial infarction was 0.2% in the ICIs plus AIs arm and 0.4% in the AIs arm, as reported in 2 trials (3, 16). In the only study reporting data on the specific cause of treatment discontinuation due to AEs (13), 2.8% patients discontinued treatment for cardiovascular AEs in the ICIs + AIs arm compared to 0% in the AIs arm. Treatment-related deaths occurred with an incidence of 1.1% for ICIs + AIs arm and 0.9% for AIs arm. Incidence of cardiovascular deaths was 0.2% in both arms.

### 3.3 Overall toxicity

The incidence of any grade and severe AEs was not significantly increased among patients treated with ICIs plus AIs compared with those treated with AIs (any grade AEs, 97.1% vs. 96.9%, OR 1.15, 95% CI: 0.71–1.85, *p* = 0.57; severe AEs, 69.7% vs. 67.2%, OR 1.18, 95% CI: 0.96–1.44, *p* = 0.11) (Supplementary Figures S1A and B). As expected, the incidence of irAEs were significantly increased among patients receiving ICIs plus AIs compared with AIs, both any grade (59.2% vs. 40.3%, OR 1.97, 95% CI: 1.21–3.21, *p* = 0.007) and severe grade (15.62% vs. 5.67%, OR 3.20, 95% CI: 1.41–7.29, *p* = 0.006) (Supplementary Figures S2A and B).

### 3.4 Efficacy

ORR was better in the ICIs plus AIs arm, compared with the AIs arm (54.8% vs. 38.6%, OR 2.25, 95% CI: 1.70–2.97, *p* < 0.001) (Supplementary Figure S3). Patients treated with ICIs plus AIs also achieved better PFS, as compared with those treated with AIs (HR 0.49, 95% CI: 0.39–0.63, *p* < 0.001) (Supplementary Figure S4). Overall survival data was not mature across trials; therefore, it was not included in the meta-analysis.

## 4 Discussion

Angiogenesis and immune escape are two important hallmarks of cancer (23). Both AIs and ICIs have demonstrated efficacy across different tumor types (1, 24), and may have a synergistic role in cancer treatment. Particularly, AIs may improve therapeutic efficacy of ICIs through the normalization of the abnormal tumor vessels, thus leading to increased infiltration of T-cells into tumor deposits, and eventually converting the intrinsically immunosuppressive tumor microenvironment to an immunosupportive one (25). Based on this rationale, combinations of ICIs and AIs have been actively investigated. Our meta-analysis reports increased efficacy for the combination of ICIs plus AIs compared with AIs alone, in terms of ORR and PFS, across several tumor types (RCC, NSCLC, HCC, CRC, ovarian and cervical cancer).

However, ICIs and AIs have different and peculiar toxicity profiles. AIs are associated with an increased risk of hypertension and cardiovascular events, including heart failure, MI, stroke, and PE (8, 26). The mechanisms underlying cardiovascular toxicity of AIs include the inhibition of vasodilation by reducing nitric oxide (NO) levels, the induction of arteriolar vasoconstriction by increasing production of endothelin 1, and the development of kidney damage through capillary rarefaction and reduction of neovascularization (27, 28). All these events may contribute to the development of hypertension, with subsequent increased risk of congestive heart failure (29). Moreover, endothelial disfunction induced by AIs may lead to the exposure of the underlying collagen, favoring the activation of coagulative cascade and the development of thrombosis (30).

ICIs are associated with irAEs in any organ or system through several and still not completely understood mechanisms, including depletion of T-regs, epitope sharing, epitope spreading, and direct toxicity, that ultimately lead to immune homeostasis dysregulation and loss of tolerance (31). Although skin, endocrine, gastrointestinal, and pulmonary are the most frequently observed irAEs, also cardiovascular irAEs have been reported with ICIs (2–4). Any part of cardiovascular system can be involved by irAEs, including blood vessels. At this regard, there is growing evidence suggesting that ICIs may contribute to the progression and inflammation of atherosclerotic plaque, thus increasing the risk for acute vascular events (5). Particularly, in a large, matched cohort retrospective study, a higher risk for acute vascular events including MI, need for coronary revascularization, and ischemic stroke, was observed among cancer patients treated with ICIs compared with those not treated with ICIs (32).

Since both ICIs and AIs may cause cardiovascular AEs, there is some concern that their combination can be synergistic for cardiovascular toxicity. In fact, in a retrospective study on NSCLC, patients treated with a combination of ICIs plus AIs had higher risk of major adverse cardiac events defined as a composite of cardiovascular death, non-fatal myocardial infarction, non-fatal stroke, and hospitalization for heart failure, when compared with patients receiving ICIs alone (HR: 2.15; 95% CI: 1.05–4.37; *p* = 0.04) (9).

In our meta-analysis, we found an increased risk for severe hypertension with the addition of ICIs to AIs. Consistently with our finding, a recently published meta-analysis of 9 articles reporting data from 8 studies (RCC = 6, HCC = 2) that included 2,833 patients treated with AIs and 2,873 patients treated with ICIs plus AIs, also showed an increased risk for high-grade hypertension (33) As reported above, hypertension is a typical AE of AIs, but data on its association with ICIs are conflicting. In a meta-analysis on 32 randomized clinical trials with a total of 19,810 cancer patients, ICIs initiation was not associated with hypertension (34). However, it is possible that hypertension could have been under-reported in clinical trials with ICIs, since it was not considered as an irAE. Recent evidence suggest that ICIs may contribute to blood pressure elevation, particularly when they are used in combination with other ICIs or other drugs. In a retrospective study on 258 melanoma patients treated ICIs, there was no significant change in systolic or diastolic blood pressure at 2 years compared with baseline. However, those treated with a combination of ICIs (ipilimumab plus nivolumab), reported a statistically significant increase (5.5 mmHg) of the systolic blood pressure (128.2 vs. 133.7 mmHg, *p* = 0.011) (35). Interestingly, in a meta-analysis of 50 trials, although ICIs alone did not increase the risk for hypertension when compared with placebo or chemotherapy, the combination of ICIs and chemotherapy significantly increased the risk of all-grade hypertension (OR = 1.34, 95% CI: 1.02–1.77, *P* = 0.04) and grade 3–5 hypertension (OR = 1.54, 95% CI: 1.10–2.15, *P* = 0.01) (36). The mechanisms underlying the occurrence of high-grade hypertension in patients treated with ICIs plus AIs are largely unknown, although it could be hypothesized a “multiple hit” theory, according to which some anticancer drugs, such as chemotherapy or AIs, may promote endothelial dysfunction, while ICIs may maintain a proinflammatory state, thus leading to vascular remodeling and consequently contributing to hypertension (37).

In our meta-analysis, we did not find an increased risk for acute vascular events, defined as stroke, myocardial infarction, and pulmonary embolism (PE), with the addition of ICIs to AIs. However, this finding should be interpreted with caution. The incidence of acute vascular events reported across the trials was low, and this could have limited the possibility to detect any significant difference among arms. Cardiovascular events are frequently not reported among clinical trials supporting contemporary anticancer therapies (38). The low number of reported events among studies with ICIs plus AIs may reflect the fact that patients enrolled in those trials were highly selected, with a low incidence of cardiovascular risk factors or pre-existing cardiovascular disease. At this regard, it is possible that well-collected real-life data in less selected populations could help to refine the cardiovascular safety profile of ICIs plus AIs combinations. Moreover, most trials did not include a standardized method of monitoring and reporting of the cardiovascular AEs, which therefore could have been under-reported. The only randomized trial of ICIs plus AIs to include prospective serial cardiac monitoring was the JAVELIN 101 Renal trial (17). Results of the cardiac monitoring have been recently published, including the incidence of major cardiovascular adverse events (MACEs), defined as grade ≥ 3 cardiovascular AEs of cardiac deaths, fatal stroke, nonfatal myocardial infarction, nonfatal congestive heart failure, nonfatal myocarditis, nonfatal arrhythmia, and nonfatal stroke (39). In this study, incidence of MACEs was higher in the ICI plus AI arm as compared with the AI arm (7.1% vs. 3.9%), and this difference could not be attributed to higher hypertension rates in the combination arm (52.1% vs. 39%), because MACE rates were similar in patients with or without hypertension (7.5% vs. 6.8%, respectively) (39). It should be considered that the definitions of acute vascular events in the present meta-analysis and that of MACEs in the JAVELIN 101 Renal trial only partially overlap.

Of note, in contrast with our results, another meta-analysis published by Crocetto et al. on the cardiovascular adverse events of ICIs plus AIs, despite the low incidence of cardiovascular toxicity different from hypertension, reported higher odds of cardiac disorders (any grade and grade 3–4), arterial thromboembolic events (any grade and grade 3–4), grade 3–4 arterial thromboembolic events and venous thrombotic events for patients receiving AIs plus ICIs compared to AIs alone (33). Conflicting results may be explained by the different studies included in, and/or by the different definitions of cardiovascular toxicities assessed by the two meta-analyses.

Therefore, although we did not find an increased risk for acute vascular events, results from the cardiac monitoring within the JAVELIN 101 Renal trial and the meta-analysis published by Crocetto et al. suggest that severe cardiovascular toxicity may be higher with ICIs plus AIs combination than with AIs alone.

Our meta-analysis has several limitations. First, it is very heterogeneous in terms of tumor types and treatments; second, the AIs included in the control arms often differ from those included in the experimental arms, thus it may be unclear whether the difference observed in terms of high-grade hypertension is due to difference in the safety profile of the AIs in the two arms, rather than their combination with ICIs in the experimental arms; finally, most trials did not report the baseline cardiovascular risk factors of patients and, as previously discussed, lack a pre-planned and standardized method of monitoring and reporting of the cardiovascular AEs.

In conclusion, results from the present meta-analysis show that the combination of ICIs and AIs increases the risk for severe hypertension and any grade irAEs, but it is more effective than AIs alone.

## Data Availability

The original contributions presented in the study are included in the article/Supplementary Material, further inquiries can be directed to the corresponding author.

## References

[1] Mellman I Coukos G Dranoff G. Cancer immunotherapy comes of age. Nature. (2011) 480(7378):480–9. 10.1038/nature10673 22193102 PMC3967235

[2] Inno A Metro G Bironzo P Grimaldi AM Grego E Di Nunno V Pathogenesis, clinical manifestations and management of immune checkpoint inhibitors toxicity. Tumori. (2017) 103(5):405–21. 10.5301/tj.5000625 28497847

[3] Inno A Tarantini L Parrini I Spallarossa P Maurea N Bisceglia I Cardiovascular effects of immune checkpoint inhibitors: more than just myocarditis. Curr Oncol Rep. (2023) 25(7):743–51. 10.1007/s11912-023-01411-7 37017825

[4] Hu JR Florido R Lipson EJ Naidoo J Ardehali R Tocchetti CG Cardiovascular toxicities associated with immune checkpoint inhibitors. Cardiovasc Res. (2019) 115(5):854–68. 10.1093/cvr/cvz026 30715219 PMC6452314

[5] Inno A Chiampan A Lanzoni L Verzè M Molon G Gori S. Immune checkpoint inhibitors and atherosclerotic vascular events in cancer patients. Front Cardiovasc Med. (2021) 8:652186. 10.3389/fcvm.2021.652186 34124192 PMC8193098

[6] Kon E Benhar I. Immune checkpoint inhibitor combinations: current efforts and important aspects for success. Drug Resist Updat. (2019) 45:13–29. 10.1016/j.drup.2019.07.004 31382144

[7] Chen DS Mellman I. Oncology meets immunology: the cancer-immunity cycle. Immunity. (2013) 39(1):1–10. 10.1016/j.immuni.2013.07.012 23890059

[8] Abdel-Qadir H Ethier JL Lee DS Thavendiranathan P Amir E. Cardiovascular toxicity of angiogenesis inhibitors in treatment of malignancy: a systematic review and meta-analysis. Cancer Treat Rev. (2017) 53:120–7. 10.1016/j.ctrv.2016.12.002 28104567

[9] Chitturi KR Xu J Araujo-Gutierrez R Bhimaraj A Guha A Hussain I Immune checkpoint inhibitor-related adverse cardiovascular events in patients with lung cancer. JACC CardioOncol. (2019) 1(2):182–92. 10.1016/j.jaccao.2019.11.013 34396181 PMC8352266

[10] Moher D Liberati A Tetzlaff J Altman DG; PRISMA Group. Preferred reporting items for systematic reviews and meta-analyses: the PRISMA statement. Br Med J. (2009) 339:b2535. 10.1136/bmj.b2535 21603045 PMC3090117

[11] Choueiri TK Powles T Burotto M Escudier B Bourlon MT Zurawski B Nivolumab plus cabozantinib versus sunitinib for advanced renal-cell carcinoma. N Engl J Med. (2021) 384(9):829–41. 10.1056/NEJMoa2026982 33657295 PMC8436591

[12] Colombo N Dubot C Lorusso D Caceres MV Hasegawa K Shapira-Frommer R Pembrolizumab for persistent, recurrent, or metastatic cervical cancer. N Engl J Med. (2021) 385(20):1856–67. 10.1056/NEJMoa2112435 34534429

[13] Finn RS Qin S Ikeda M Galle PR Ducreux M Kim TY Atezolizumab plus bevacizumab in unresectable hepatocellular carcinoma. N Engl J Med. (2020) 382(20):1894–905. 10.1056/NEJMoa1915745 32402160

[14] McDermott DF Huseni MA Atkins MB Motzer RJ Rini BI Escudier B Clinical activity and molecular correlates of response to atezolizumab alone or in combination with bevacizumab versus sunitinib in renal cell carcinoma. Nat Med. (2018) 24(6):749–57. 10.1038/s41591-018-0053-3 29867230 PMC6721896

[15] Mettu NB Ou FS Zemla TJ Halfdanarson TR Lenz HJ Breakstone RA Assessment of capecitabine and bevacizumab with or without atezolizumab for the treatment of refractory metastatic colorectal cancer: a randomized clinical trial. JAMA Netw Open. (2022) 5(2):e2149040. 10.1001/jamanetworkopen.2021.49040 35179586 PMC8857687

[16] Moore KN Bookman M Sehouli J Miller A Anderson C Scambia G Atezolizumab, bevacizumab, and chemotherapy for newly diagnosed stage III or IV ovarian cancer: placebo-controlled randomized phase III trial (IMagyn050/GOG 3015/ENGOT-OV39). J Clin Oncol. (2021) 39(17):1842–55. 10.1200/JCO.21.00306 33891472 PMC8189598

[17] Motzer RJ Robbins PB Powles T Albiges L Haanen JB Larkin J Avelumab plus axitinib versus sunitinib in advanced renal cell carcinoma: biomarker analysis of the phase 3 JAVELIN renal 101 trial. Nat Med. (2020) 26(11):1733–41. 10.1038/s41591-020-1044-8 32895571 PMC8493486

[18] Motzer R Alekseev B Rha SY Porta C Eto M Powles T Lenvatinib plus pembrolizumab or everolimus for advanced renal cell carcinoma. N Engl J Med. (2021) 384(14):1289–300. 10.1056/NEJMoa2035716 33616314

[19] Rini BI Plimack ER Stus V Gafanov R Hawkins R Nosov D Pembrolizumab plus axitinib versus sunitinib for advanced renal-cell carcinoma. N Engl J Med. (2019) 380(12):1116–27. 10.1056/NEJMoa1816714 30779529

[20] Rini BI Powles T Atkins MB Escudier B McDermott DF Suarez C Atezolizumab plus bevacizumab versus sunitinib in patients with previously untreated metastatic renal cell carcinoma (IMmotion151): a multicentre, open-label, phase 3, randomised controlled trial. Lancet. (2019) 393(10189):2404–15. 10.1016/S0140-6736(19)30723-8 31079938

[21] Socinski MA Jotte RM Cappuzzo F Orlandi F Stroyakovskiy D Nogami N Atezolizumab for first-line treatment of metastatic nonsquamous NSCLC. N Engl J Med. (2018) 378(24):2288–301. 10.1056/NEJMoa1716948 29863955

[22] Sugawara S Lee JS Kang JH Kim HR Inui N Hida T Nivolumab with carboplatin, paclitaxel, and bevacizumab for first-line treatment of advanced nonsquamous non-small-cell lung cancer. Ann Oncol. (2021) 32(9):1137–47. 10.1016/j.annonc.2021.06.004 34139272

[23] Hanahan D Weinberg RA. Hallmarks of cancer: the next generation. Cell. (2011) 144(5):646–74. 10.1016/j.cell.2011.02.013 21376230

[24] Patel SA Nilsson MB Le X Cascone T Jain RK Heymach JV. Molecular mechanisms and future implications of VEGF/VEGFR in cancer therapy. Clin Cancer Res. (2023) 29(1):30–9. 10.1158/1078-0432.CCR-22-1366 35969170 PMC10274152

[25] Fukumura D Kloepper J Amoozgar Z Duda DG Jain RK. Enhancing cancer immunotherapy using antiangiogenics: opportunities and challenges. Nat Rev Clin Oncol. (2018) 15(5):325–40. 10.1038/nrclinonc.2018.29 29508855 PMC5921900

[26] Kamba T McDonald DM. Mechanisms of adverse effects of anti-VEGF therapy for cancer. Br J Cancer. (2007) 96(12):1788–95. 10.1038/sj.bjc.6603813 17519900 PMC2359962

[27] Böhm F Pernow J. The importance of endothelin-1 for vascular dysfunction in cardiovascular disease. Cardiovasc Res. (2007) 76(1):8–18. 10.1016/j.cardiores.2007.06.004 17617392

[28] Mourad JJ des Guetz G Debbabi H Levy BI. Blood pressure rise following angiogenesis inhibition by bevacizumab. A crucial role for microcirculation. Ann Oncol. (2008) 19(5):927–34. 10.1093/annonc/mdm550 18056916

[29] Nazer B Humphreys BD Moslehi J. Effects of novel angiogenesis inhibitors for the treatment of cancer on the cardiovascular system: focus on hypertension. Circulation. (2011) 124(15):1687–91. 10.1161/CIRCULATIONAHA.110.992230 21986775

[30] Di Lisi D Madonna R Zito C Bronte E Badalamenti G Parrella P Anticancer therapy-induced vascular toxicity: VEGF inhibition and beyond. Int J Cardiol. (2017) 227:11–7. 10.1016/j.ijcard.2016.11.174 27866063

[31] Pauken KE Dougan M Rose NR Lichtman AH Sharpe AH. Adverse events following cancer immunotherapy: obstacles and opportunities. Trends Immunol. (2019) 40(6):511–23. 10.1016/j.it.2019.04.002 31053497 PMC6527345

[32] Drobni ZD Alvi RM Taron J Zafar A Murphy SP Rambarat PK. Association between immune checkpoint inhibitors with cardiovascular events and atherosclerotic plaque. Circulation. (2020) 142(24):2299–311. 10.1161/CIRCULATIONAHA.120.049981 33003973 PMC7736526

[33] Crocetto F Ferro M Buonerba C Bardi L Dolce P Scafuri L Comparing cardiovascular adverse events in cancer patients: a meta-analysis of combination therapy with angiogenesis inhibitors and immune checkpoint inhibitors versus angiogenesis inhibitors alone. Crit Rev Oncol Hematol. (2021) 188:104059. 10.1016/j.critrevonc.2023.104059 37353178

[34] Minegishi S Kinguchi S Horita N Namkoong H Briasoulis A Ishigami T Immune checkpoint inhibitors do not increase short-term risk of hypertension in cancer patients: a systematic literature review and meta-analysis. Hypertension. (2022) 79(11):2611–21. 10.1161/HYPERTENSIONAHA.122.19865 36093785

[35] Turker I Sharma A Huang S Johnson DB Alexander MR. Combination immune checkpoint inhibitor therapy is associated with increased blood pressure in melanoma patients. Hypertension. (2023) 80(3):e43–5. 10.1161/HYPERTENSIONAHA.122.20407 36398660 PMC9931662

[36] Liu S Gao W Ning Y Zou X Zhang W Zeng L Cardiovascular toxicity with PD-1/PD-L1 inhibitors in cancer patients: a systematic review and meta-analysis. Front Immunol. (2022) 13:908173. 10.3389/fimmu.2022.908173 35880172 PMC9307961

[37] Gallo G Volpe M Savoia C. Endothelial dysfunction in hypertension: current concepts and clinical implications. Front Med (Lausanne). (2022) 8:798958. 10.3389/fmed.2021.798958 35127755 PMC8811286

[38] Bonsu JM Guha A Charles L Yildiz VO Wei L Baker B Reporting of cardiovascular events in clinical trials supporting FDA approval of contemporary cancer therapies. J Am Coll Cardiol. (2020) 75(6):620–8. 10.1016/j.jacc.2019.11.059 32057377 PMC7860639

[39] Rini BI Moslehi JJ Bonaca M Schmidinger M Albiges L Choueiri TK Prospective cardiovascular surveillance of immune checkpoint inhibitor-based combination therapy in patients with advanced renal cell cancer: data from the phase III JAVELIN renal 101 trial. J Clin Oncol. (2022) 40(17):1929–38. 10.1200/JCO.21.01806 35239416 PMC9177241

