# Supplementary material for: Risk of cardiovascular toxicity with combination of immune-checkpoint inhibitors and angiogenesis inhibitors: a meta-analysis
Source: Front Cardiovasc Med. 2024 Feb 2;11:1309100. doi: 10.3389/fcvm.2024.1309100 (PMC10869562; doi:10.3389/fcvm.2024.1309100)
Supplement: Supplementary file 1 [file Table1.docx]

Supplementary Material

# Supplementary Figures


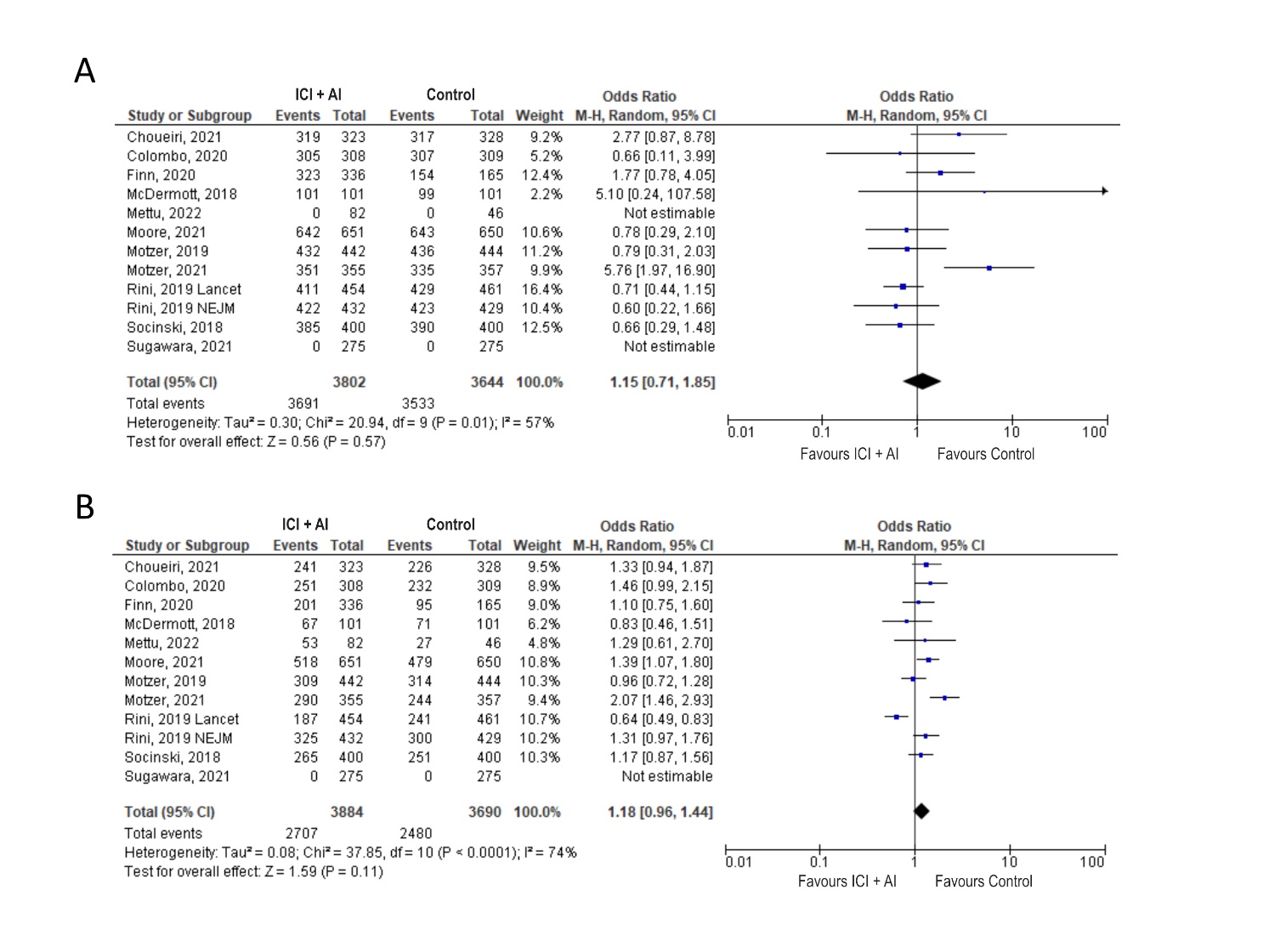


**Supplementary Figure 1**. Forest plot showing odds ratio for all reported AEs **(A)** and severe AEs (grade ≥ 3) **(B)** for the 12 studies that explicitly reported the total number of AEs and severe AEs, respectively, by ICIs + AIs vs control. The risk ratio for each adverse event is represented by a square, and the horizontal lines crossing the squares represent the 95% confidence interval (CI).


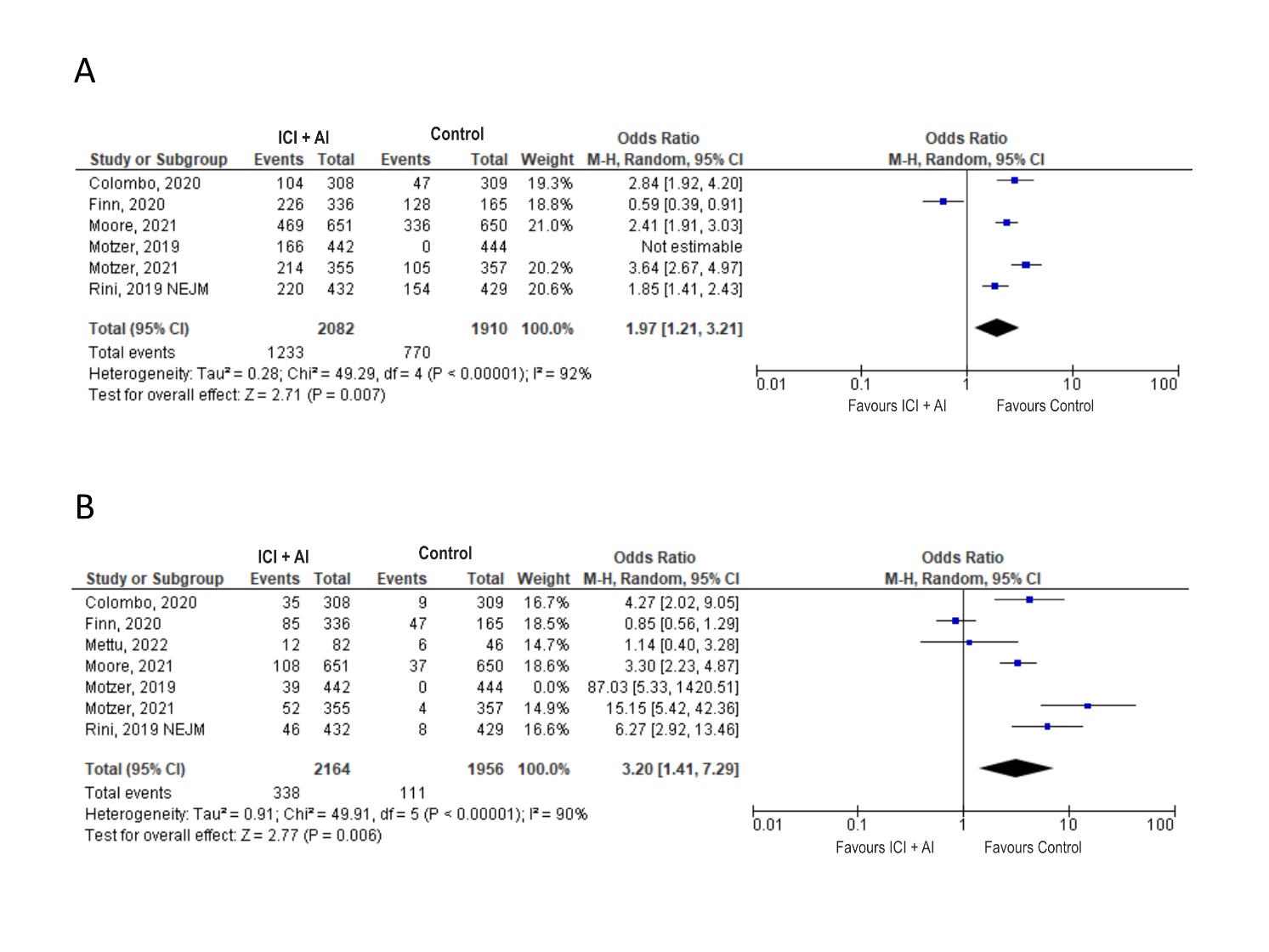


**Supplementary Figure 2.** Forest plot showing odds ratio for all reported irAEs **(A)** and severe irAEs (grade ≥ 3) **(B)** for the studies that explicitly reported the total number of irAEs and severe irAEs, respectively, by ICIs + AIs vs control. The risk ratio for each adverse event is represented by a square, and the horizontal lines crossing the squares represent the 95% confidence interval (CI).


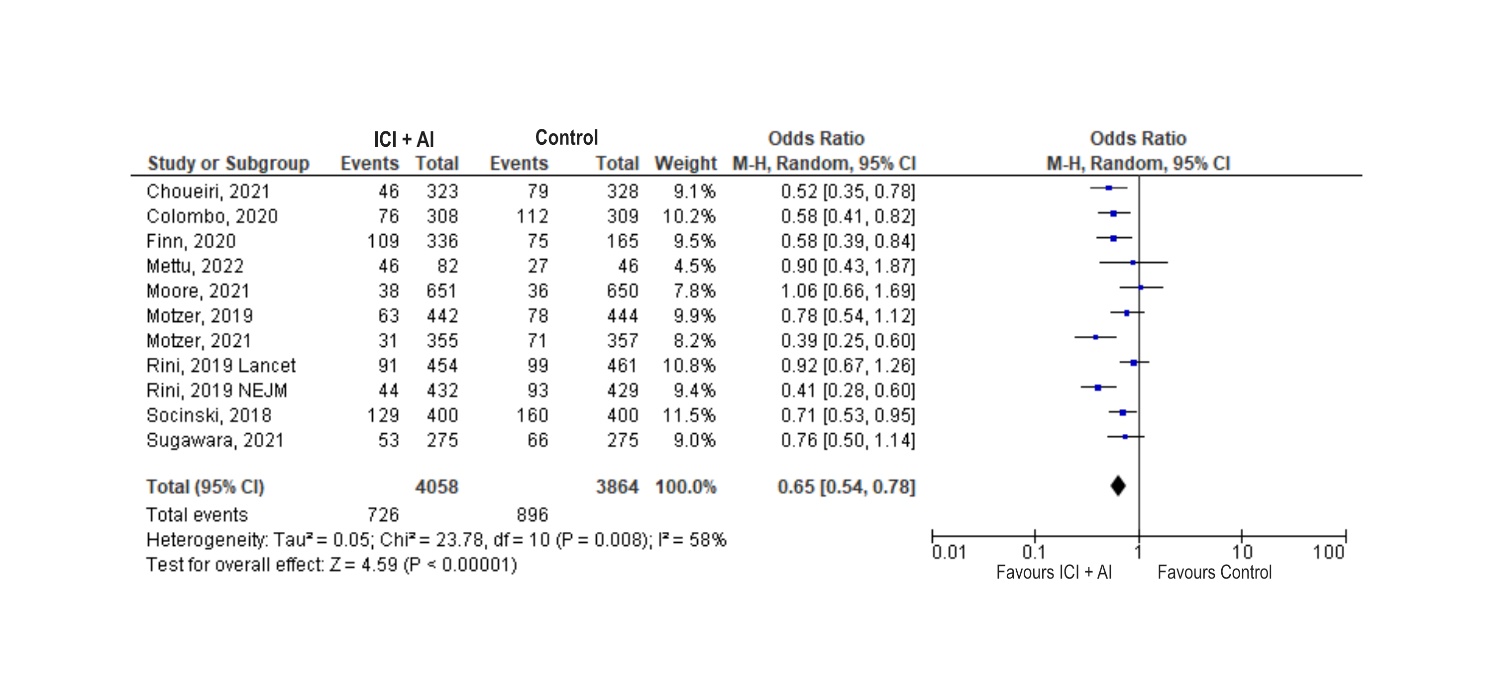


**Supplementary Figure 3.** Forest plot showing odds ratio for 1-year ORR for the 12 studies that compared ORR of ICIs + AIs vs control.


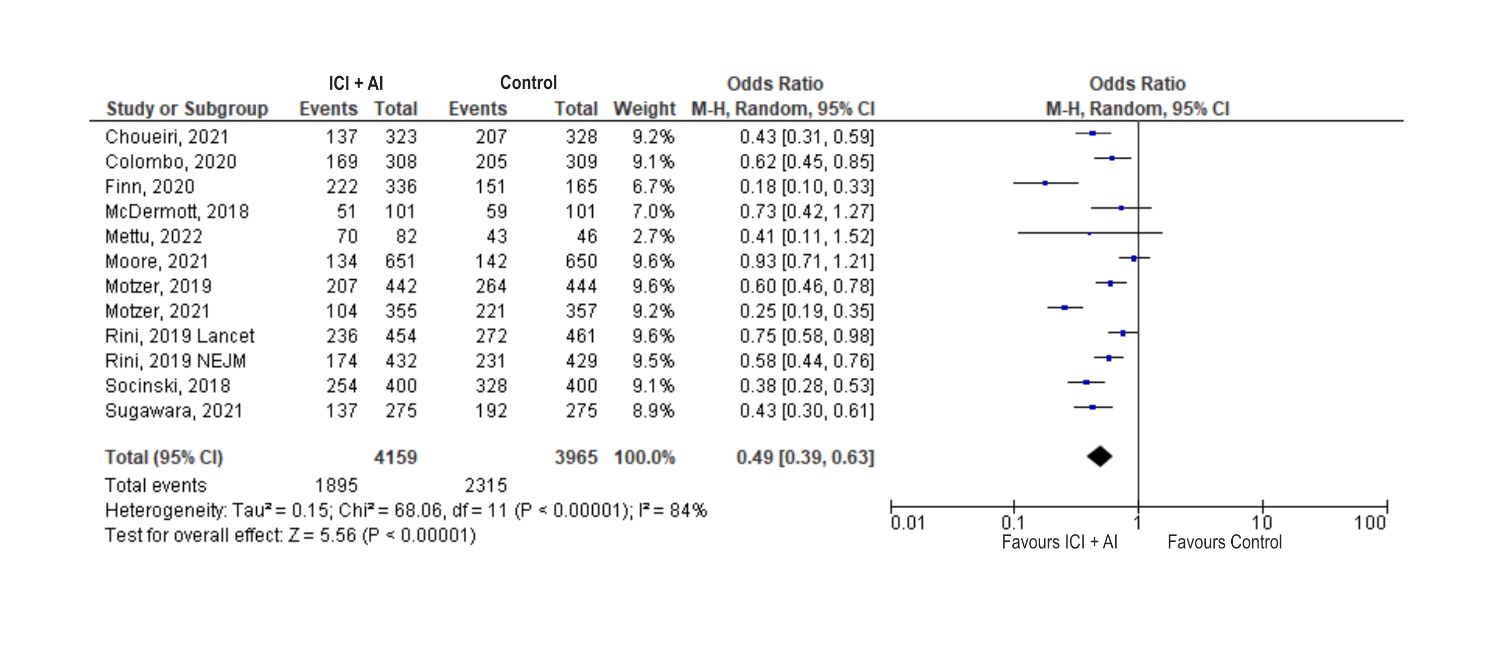
 **Supplementary Figure 4.** Forest plot showing odds ratio for 1-year PFS for the 12 studies that compared PFS events of ICIs + AIs vs control.
